# Rapid qualitative review of ethical issues surrounding healthcare for pregnant women or women of reproductive age in epidemic outbreaks

**DOI:** 10.4178/epih.e2018003

**Published:** 2018-01-23

**Authors:** Patrik Hummel, Abha Saxena, Corinna Klingler

**Affiliations:** 1 Department of Philosophy, University of St Andrews, St Andrews, United Kingdom; 2 Ethics and Knowledge, World Health Organization, Geneva, Switzerland; 3 Institute of Ethics, History and Theory of Medicine, Ludwig-Maximilians-University Munich, Munich, Germany

**Keywords:** Ethics, Pregnancy, Epidemics, Reproductive health, Review

## Abstract

This article describes, categorizes, and discusses the results of a rapid literature review aiming to provide an overview of the ethical issues and corresponding solutions surrounding pregnancies in epidemic outbreaks. The review was commissioned by the World Health Organization to inform responses to the Zika outbreak that began in 2015. Due to the urgency of the response efforts that needed to be informed by the literature search, a rapid qualitative review of the literature published in PubMed was conducted. The search and analysis were based on the operationalization of 3 key concepts: ethics, pregnancy, and epidemic outbreak. Ethical issues and solutions were interpreted within a principlist framework. The data were analyzed using qualitative content analysis. The search identified 259 publications, of which the full text of 23 papers was read. Of those, 20 papers contained a substantive part devoted to the topic of interest and were therefore analyzed further. We clustered the ethical issues and solutions around 4 themes: uncertainty, harms, autonomy/liberty, and effectiveness. Recognition of the identified ethical issues and corresponding solutions can inform and improve response efforts, public health planning, policies, and decision-making, as well as the activities of medical staff and counselors who practice before, during, or after an epidemic outbreak that affects pregnant women or those of reproductive age. The rapid review format proved to be useful despite its limited data basis and expedited review process.

## INTRODUCTION

In spring 2015, Brazil witnessed an outbreak of the Zika virus. Despite efforts to control the virus, it continues to spread through the Americas, parts of Oceania and the Pacific Islands, as well as Cape Verde [1]. In 2016 and 2017, 66 countries reported Zika transmission [2]. Zika is primarily transmitted by *Aedes* mosquitoes. Cases of perinatal and sexual transmission have been reported as well. Reports of transmission through blood transfusion are being investigated [1]. In adults, Zika normally causes mild fever, joint pain, rashes, conjunctivitis, or no symptoms at all. Although the Zika virus has been known since the 1940s, new public health challenges are being raised by the current large-scale outbreak. Concerns arose that Zika can cause Guillain-Barré syndrome and a range of congenital disorders, most notably microcephaly [3,4]. While the likelihood and precise mechanisms of these effects are still being investigated, their potential severity caused increased attention to be paid to the management of ethical issues, such as the appropriate implementation of surveillance activities for improving the evidence base [5]. The outbreak continues to present policy-makers with the challenge of deciding on ethically adequate public health responses.

When dealing with these issues, it seems advisable to consider experiences acquired in previous epidemics. Overviews of ethical issues and the management strategies that have been implemented allow us to transfer insights to the new epidemic. Systematic literature reviews can help in providing such comprehensive overviews. Systematic reviews on normative issues are still rare [6], but some have been conducted in areas such as dementia care [7], patient safety research [8], and public health surveillance [9]. Since no adequate overviews of ethical issues for the present context existed, the World Health Organization (WHO) commissioned a qualitative literature review in support of the development and refinement of Zika response efforts. Since pregnant women were anticipated to be affected in ways that might bring about ethically challenging scenarios, the following 2 research questions were formulated:

Research question 1: What are the ethical issues surrounding healthcare for pregnant women that arise in the context of epidemic outbreaks?
Research question 2: Which steps should be taken to mitigate or solve ethical issues that fall under research question 1?

The objective of this literature review was thus to identify relevant ethical issues and solutions. Being aware of them will facilitate and improve public health response efforts.

## METHODS

The methodology of qualitative systematic reviews on normative questions is evolving, and no clear guidelines have yet been developed. The reporting of methods follows the PRISMA (Preferred Reporting Items for Systematic Reviews and Meta-Analyses) statement as far as applicable to qualitative evidence syntheses [10].

Due to the urgency of the response efforts for which this review was conducted, we were only able to perform a rapid review and accordingly limited the search to PubMed. We identified 3 central concepts contained in the research questions: ‘ethical issue,’ ‘epidemic outbreak,’ and ‘pregnancy.’ Synonyms of those words and similar terms were used to build the search strategy (Table 1). The search was conducted in February 2016.

We operationalized these terms in order to formulate criteria for the inclusion and exclusion of publications in the review. As in other systematic qualitative reviews conducted on normative questions [7,9,11], we understood ‘ethical issue’ in a principlist [12] sense as either the ‘risk’ of not satisfying an ethical principle or a ‘conflict’ between 2 ethical principles. Since we were also interested in identifying management strategies, we also targeted safeguards for preventing a risk from materializing and conditions that justify solving a conflict between ethical principles. We chose ‘solution’ as an umbrella term for these safeguards and conditions. In particular, we were guided by the framework developed by Marckmann et al. [13], which identifies beneficence, non-maleficence, respect for autonomy, equity, and efficiency as *prima facie* binding and action-guiding principles. There are further public health ethics frameworks [14] that we could have used, but we chose this one as it builds on experiences with previously published (principlist) frameworks and is rather inclusive with regard to ethical principles. ‘Epidemic outbreak’ was understood as an unexpected temporal increase in the case numbers of a given disease in a certain country or region [15]. We opted to understand ‘pregnancy’ in a wide sense as covering topics concerning not only the process preceding birth, but also women of reproductive age who might become pregnant.

Publications were included if they addressed research questions 1 or 2 by discussing ‘ethical issue[s]’, ‘epidemic outbreak[s]’, and ‘pregnancy’ as defined above. Moreover, discussion of these topics had to make up a considerable part of the publication rather than being limited to quasi-incidental findings. If the topics were only mentioned or addressed in passing, the publication was excluded. In addition, only publications in English were included. PH screened the title and abstract of all identified articles. For papers identified as possibly relevant, we sought access via 3 university libraries. Authors were additionally contacted to gain access to publications where contact details were available. Data were analyzed and synthesized by means of an adapted version of qualitative content analysis [16]. Our framework providing an overview of ethical issues and solutions was developed inductively by means of the strategies of progressively summarizing and subsumption [16]. PH read the full text of the selected papers, extracted relevant quotes, and summarized and subsumed the ethical issues and solutions articulated in the publications. CK provided detailed comments in writing and during discussions to ensure plausibility, consistency, and soundness at several stages of the selection and synthesis process.

## RESULTS

**Figure 1.**
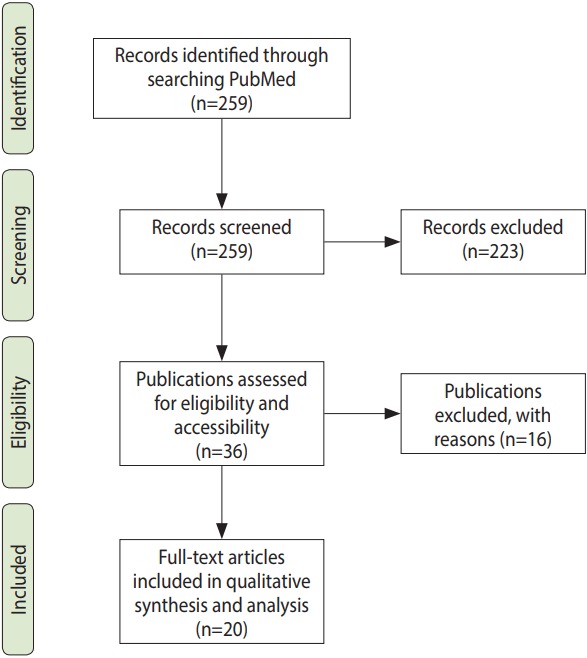
Screening process. Adapted from Moher D, et al. Ann Intern Med 2009;151:264-269 [10].

Our search strategy identified 259 publications. After screening the titles and abstracts, 36 publications were identified as relevant because they matched the inclusion and exclusion criteria. Unfortunately, 13 of these 36 publications were not available to us in full text, even after contacting the authors directly. The remaining 23 publications were read, summarized, and synthesized. These were published between 1975 and 2015; 13 of them between 2008 and 2015, 7 between 1975 and 1995, and 3 between 1996 and 2007. Nine publications were primarily concerned with human immunodeficiency virus, 6 with influenza, 2 with rubella, 1 with Ebola, 1 with cancer, and 4 were not focused on a specific disease. Three publications read in full-text fell short of addressing the research questions, and were excluded. The screening process is presented in Figure 1. We provide bibliographical information for all included papers in the Supplementary Material 1.

We identified ethical issues and solutions mentioned in the publications, and clustered them around 4 themes that became salient during the summarizing and subsumption process: uncertainty, harm, autonomy/liberty, and effectiveness. An issue or solution was understood as relating to uncertainty if it concerned a lack of knowledge or guidance. It pertained to harm if it concerned any event or state of affairs that decreased the well-being of individuals, communities, or populations. The theme autonomy/liberty included issues and solutions speaking to the freedom and informedness of individual choice. Effectiveness covered issues and solutions relating to the proper functioning of interventions for controlling the outbreak or mitigating its effects. We provide examples of ethical issues in Table 2, and of proposed management strategies (accompanied by the ethical issues they address) in Table 3. Complete lists with all subcodes, as well as example quotes allowing readers to retrace our synthesis process, are available in the Supplementary Materials 2 and 3. These tables and materials should be seen as the main output of our review. However, we also would like to provide a brief narrative summary of our findings.

While the reviewed papers presented a significant number of issues relating to each theme, they mentioned relatively few solutions related to uncertainty and effectiveness. The number of solutions proposed for violations of autonomy was comparably high. Generally, a striking result was that the reviewed papers tended to discuss issues, particularly risks. The provision of solutions was much less common. Often these remained implicit.

In the reviewed papers, uncertainty regarding clinical aspects was said to the endanger diagnosis, prevention, and treatment of pregnant women. For example, there is a risk of pregnant women being insufficiently included in the research agenda, such that knowledge is lacking for clinical decision-making on treatment options and the use of investigational drugs [17]. Policy-making is complicated by uncertainty not only regarding medical facts, but also normative questions. For example, it might be unclear to what extent policies for the prevention of mother-to-child transmission may interfere with women’s reproductive freedom [18]. Different populations can be harmed by the disease itself, but also by stigmatization/discrimination and insufficient healthcare services. For example, antenatal hospital visits can increase pregnant women’s risk of exposure to the epidemic in the clinic [19]. Autonomy can be compromised by consequences of the epidemic, but also through direct or indirect interference with the (reproductive) choices of pregnant women. For example, social pressures and the tacit introduction of standards for adequate choice in counseling services can have significant effects [20]. Finally, the theme of effectiveness occurred primarily in connection with control strategies for sexually transmittable epidemics. For instance, there is a risk that a control strategy based on contraceptives may be ineffective when no adequate support, counseling, or guidance is provided [21].

The few suggested solutions related to uncertainty were mainly concerned with the implementation of adequate and timely procedures for formulating preemptive, formal preparedness plans [22]. A range of different solutions were suggested for harms. For example, during an epidemic, pregnant women should receive services independently of insurance status and purely on the basis of their clinical needs [23]. As an example of how interference with autonomy/liberty can be avoided, one author suggested that states must liberalize abortion laws and regard some infections as sufficient grounds for facilitating access to pregnancy termination services [24]. The effectiveness of epidemic control strategies for sexually transmittable diseases should be ensured by means of both condom distribution and behavioral strategies that promote, for example, faithfulness and partner reduction [21].

## DISCUSSION

### Limitations

Due to the limited timeframe for this review, we could only conduct a rapid and not a full systematic review. This means we had to make concessions regarding the breadth and rigor of the analysis. First, we only searched PubMed for the relevant literature and therefore could only include scientific journal publications. Searching book publications via Google Books or the gray literature (including policy reports) might have provided us with additional insights. We were furthermore limited to the literature published in English. Nonetheless, no publications were excluded on this basis. Second, only 1 person (PH) conducted the screening and analysis process of individual papers, in which 2 or more people are normally involved. However, a second person (CK) was approached where uncertainties arose during screening and extraction. Furthermore, the process of synthesizing the data to obtain the final framework was done in a discursive process between PH and CK. Reviews of normative questions always involve a high level of interpretation because authors do not always clearly describe issues at hand, and there are various justifiable ways of synthesizing the data in a comprehensible format. We think that our approach of incorporating thorough discussions, especially during the most difficult phase of synthesizing, ensures the reliability and validity of our representation of the findings.

A more general limitation that is not related to the rapid format of this review is our operationalization of the fundamental concepts on which the search was based, especially ‘ethical issues.’ We admit that other definitions might have given slightly different outcomes. However, we had good reasons for choosing a principlist understanding of ethical issues, primarily because it is the most commonly used framework in public health ethics. Moreover, other systematic reviews of normative information have already demonstrated the instrumental value of the principlist approach for a descriptive and stakeholder-oriented analysis.

Moreover, ethical issues might arise that are not captured by this literature search because they are not discussed at all in the literature. It might very well be that certain issues are not on the radar of those with a scholarly interest in public health (ethics), but still cause severe trouble in the field. This review should therefore only be seen as a starting point and not as a full list of all potential issues and solutions. Further steps such as discussions with stakeholders are therefore suggested to get a full overview of ethical issues in this context.

### Reflections on the rapid review format

Epidemic outbreaks have the potential to affect a large number of individuals. Policy-makers need to ensure that responses are ethically sound and therefore acceptable to those affected. Since such outbreaks normally require relatively urgent responses, an informative yet time-efficient tool is needed to inform these efforts. The rapid review format is useful in new contexts such as epidemic outbreaks of unknown or rare epidemic diseases, outbreaks that are much larger in scale than previous ones, and emerging and pressing medical and public health phenomena in general. In each of these cases, one can expect that even if the target phenomenon exhibits a significant degree of novelty, some of the challenges that arise will be partly familiar from previous public health contexts. At the very least, it seems warranted to look for such similarities and take existing knowledge and evidence into account in order to ensure that lessons from past challenges are drawn and considered. The rapid review format is a time- and resource-efficient way to look back in order to plan ahead. Although the process of screening the existing literature in the target domain is expedited, the insights can contribute to the soundness and effectiveness of timely response efforts. In our case, fewer than 6 months passed from the initiation of the project to the submission of the final report. If the authors had been able to work full-time on the review, it would have been finalized even faster. This makes the rapid review format a useful tool for epidemic outbreaks where severe time constraints and urgency require decisions before a systematic review can be carried out. Despite the rapid execution and narrowed data base, the rapid review format likely offers a reasonable compromise between time-efficiency and the completeness of a full but more time-consuming systematic review. It remains an exciting question for further research exactly how attractive this trade-off is, and to what extent informativeness and completeness are inevitably compromised by the concessions.

Furthermore, our review was descriptive, and did not evaluate the considerations that were identified. This means that only mentioning issues and solutions, not the quality or soundness thereof, was sufficient for inclusion. In contrast to systematic reviews that try to answer quantitative, empirical questions for which frameworks such as GRADE for evaluating the quality of evidence and strength of recommendations [25] exist, there are no widely accepted standards for evaluating normative considerations. Accordingly, no analogues to the quality assessment criteria that are standard in systematic reviews on quantifiable topics exist. Moreover, no attempt was made to evaluate whether the identified considerations were relevant to the context of Zika. As mentioned, the reviewed papers discussed a range of different diseases. Whether these diseases and outbreaks share relevant similarities with the current Zika outbreak and render the identified considerations applicable is beyond the scope of our review. Addressing this question requires in-depth understanding of the disease itself, as well as a thorough knowledge of the national and local circumstances in which it occurs. For example, the risk that pregnancy complications and infections with the epidemic disease are hard to distinguish because they present similar symptoms [19] does not seem applicable to Zika, given what is known about the symptoms caused by the virus. Further reflection and discussion on the issues and solutions that we identified, as well as on the specific context of the present outbreak, is necessary before the findings can facilitate decision-making.

### Outlook on Zika

With these caveats in mind, we offer below some tentative remarks that go beyond mere statements of our findings, and concern the relationship between the identified issues and solutions and the Zika outbreak that prompted our review.

Some of the issues that we identified have materialized in the Zika outbreak, such as the risk that there might be limited evidence on the effects of the epidemic on pregnant women and fetuses (in our review: [26]). In early 2016, the WHO [27] could speak only of a possible association between the virus and congenital malformations and stressed how many open questions remain.

Moreover, a paper noted that recommendations on reproductive choices during an outbreak could be too directive and infringe upon individuals’ autonomy (in our review: [18]). Indeed, some have argued that the June 2016 recommendation of the WHO that individuals in affected areas “be correctly informed and oriented to consider delaying pregnancy” [28] is more than a merely descriptive and non-directional piece of information, but actually directs individuals to an ethically relevant extent [29]. This is not to say that the recommendation is indeed ethically problematic, only that its justifiability has been drawn into question and might require further ethical reflection.

As a final example, our review captured the concern that inconsistent access to contraceptives and reproductive health services (in our review: [24]) can render recommendations and individuals’ decisions ineffective. Correspondingly, it is suspected that capacities, infrastructure, supplies, training, and restrictive abortion laws in Zika-affected countries complicate women’s access to family planning services [30].

The overlap between the risks identified in the review and those noticed in the Zika outbreak suggests at least 2 conclusions. Firstly, it validates the fit of our research questions, search strategy, and results with the real-world public health challenge. Secondly, it highlights aspects where Zika does not pose new and unique challenges, but exhibits characteristics that are familiar from other epidemics [31]. Building on knowledge acquired during preceding epidemics is therefore possible.

It is thus encouraging to see that just as some risks have materialized, some solutions from previous outbreaks have been recognized and implemented, too. For example, the Pan American Health Organization (PAHO) has made recommendations along the lines of the identified solutions. In view of the limited evidence on Zika, there is an urgent need to include pregnant women in research agendas. Otherwise, the scientific community will continue to face incomplete knowledge of the effects of Zika and a lack of diagnostics, vaccines, and/or treatment options (in our review: [17]). Pregnant women thus play a key role in PAHO’s research agenda [32].

Further overlap exists between the solutions identified in our review and those advanced in PAHO’s ethics guidelines on Zika, which recommend that pregnant women should be provided with up-to-date and understandable information on the disease and treatment options ([33]; in our review: [34]) – a demand that also figures prominently in a note from the Nuffield Council on Bioethics [5]. Women’s decisions on testing, treatment, and reproduction need to be sought and respected [23,33], Contraceptives need to be made available for protection and family planning [21,24,33], which will also allow women to follow advice to delay pregnancies.

In view of the latter considerations, it is relevant to note that one of the papers emphasized a communication by the Pope according to which condom use is not out of the question for Catholics (in our review: [21]), provided that lives are at risk and condoms help to save them. Christians can even see condom use as an act of responsibility, care, and love towards sexual partners (in our review: [21]). In case questions remained whether this reasoning translates to Zika, the Pope has spoken in favor of condom usage to slow down the outbreak and to protect women that are potentially affected by Zika [35]. Policy-makers in the predominantly Catholic countries in which the Zika outbreak is occurring should consider these recommendations when developing their public health responses.

## CONCLUSION

This article describes, categorizes, and discusses the results of a rapid literature review on ethical issues and solutions surrounding pregnancies in epidemic outbreaks. Recognition of the identified ethical issues and corresponding solutions can inform and improve response efforts, public health planning, policies, and decision-making, as well as the activities of medical staff and counselors who practice before, during, or after an epidemic outbreak that affects pregnant women.

## Figures and Tables

**Table 1.** Search strategy for PubMed

Central concept	Search strategies
Ethics	("ethics"[MeSH Terms] OR "morals"[MeSH Terms] OR "human rights"[MeSH Terms] OR "government regulation"[MeSH Terms] OR ethic*[Text Word] OR bioethic*[Text Word] OR moral*[Text Word] OR "reproductive right"[Text Word] OR "reproductive rights"[Text Word] OR "human right"[Text Word] OR "human rights"[Text Word] OR justice[Text Word] OR "Helsinki Declaration"[Text Word] OR "Hippocratic Oath"[Text Word] OR governance[Text Word]) AND
Pregnancy	("pregnancy"[MeSH Terms] OR "abortion, induced"[MeSH Terms] OR "abortion, spontaneous"[MeSH Terms] OR "abortion applicants"[MeSH Terms] OR "reproductive health services"[MeSH Terms] OR "reproductive behavior"[MeSH Terms] OR "family planning policy"[MeSH Terms] OR "maternal exposure"[MeSH Terms] OR "maternal death"[MeSH Terms] OR "fetus"[MeSH Terms] OR "fetal mortality"[MeSH Terms] OR "congenital abnormalities"[MeSH Terms] OR "maternal fetal relations"[MeSH Terms] OR pregnan*[Text Word] OR childbearing[Text Word] OR fetal[Text Word] OR foetal[Text Word] OR fetus[Text Word] OR foetus[Text Word] OR contracepti*[Text Word] OR "family planning"[Text Word] OR miscarriage[Text Word] OR abortion[Text Word] OR matern*[Text Word] OR neonat*[Text Word] OR fertility[Text Word] OR perinatal[Text Word] OR antenatal[Text Word] OR prenatal[Text Word] OR postnatal[Text Word] OR birth[Text Word] OR obstetric*[Text Word]) AND
Epidemic outbreak	(disease outbreak[MeSH Terms] OR communicable disease, emerging[MeSH Terms] OR epidemic*[Text Word] OR pandemic*[Text Word] OR outbreak*[Text Word] OR "health emergency"[Text Word] OR "health emergencies"[Text Word])

**Table 2.** Identified issues

Themes	Codes	Subcodes (examples; full list as supplementary material)
Risks of misguided judgement related to systematic uncertainty in medical decision-making	Risks related to diagnosis in pregnant women	Risk that pregnancy complications give similar symptoms as the epidemic (e.g., Ebola virus disease)
	Risks related to prevention and treatment of infected pregnant women	Risk of lacking standards or guidance on when an investigational drug can and/or should be used in pregnant women
Risks of misjudgements related to systematic uncertainty in policy decision-making and guideline development	Risks related to the appropriate design or lack of individual hospital preparedness plans	Risk that one cannot anticipate women's treatment choices in epidemics, which complicates the development of effective preparedness plans
	Risk related to lack of evidence on technical aspects	Risk that there is limited evidence on the effects of a given epidemic on pregnant women and their fetuses
	Risk related to lack of guidance/answers on normative questions	Risks related to unclarity to what extent policies for the prevention of mother-to-child transmission may influence or interfere with women's reproductive choices
Issues of harm affecting pregnant women	Issues of increased harms (mortality, morbidity) caused by insufficient access to health services in epidemics	Risk that prevention of mother-to-child transmission is focused only on the well-being of the infant, noton the mother
	Risks of increased harms (mortality, morbidity) caused by inadequate provision of health services in epidemics	Risk that during an epidemic, protective equipment makes it more difficult to deliver obstetric services safely
	Risk of harming infected mothers through stigmatization and criminalization	Risk that mandatory testing and programmes for the prevention of mother-to-child transmission do not ensure confidentiality, causing stigma and discrimination
	General risks of harm caused by pregnancy in epidemics	Risk that pregnancy can aggravate a pre-existing infection with an epidemic
Risks of harming women of reproductive age	Risks of increased harms caused by (organization of) screening in epidemics	Risk that due to privacy breaches after screening, a woman of reproductive age experiences domestic violence
	Risk of stigmatization and persecution	Risk that public discourse singles out women as sources of transmission, when in fact men are drivers in transmission, too
	Increased risk of infection for women of reproductive age	Risk that because healthcare professionals are traditionally woman, transmission risks for healthcare professionals affect women disproportionately
	Risks of men violence as a reaction to preventive measures	Risk that intra-marital sexual violence increases because the woman is perceived to fear contracting an epidemic from her partner
Issues of harming the child	Issues of infecting the child with the epidemic disease	Risk that the epidemic contributes to infections, congenital disorders, disabilities, miscarriages, etc
	General issues arising as a consequence of the epidemic	Risk that a child will be born to infected parents who will pass away or be unable to raise the child
Issues of harming healthcare professionals		Conflict between health care workers’protective rights and the public health need for their services in emergencies
Issues of harming the public/public health		Risk that infected pregnant women act as a catalyst, transmitting the epidemic to other parts of the population
Issues regarding pregnant women’s autonomous decisions being compromised	Risks of medical factors compromising autonomy or autonomous decisions	Risk that infection renders the pregnant women unable (at least temporarily) to make decisions
	Issues of direct interference by others	Risk that a pregnant woman’s choices about diagnosis and/or treatment are not respected
	Issues of indirect interference	Conflict between a woman's wish to use contraceptives vs. religious values which do not allow them
	Risks that inadequate counselling about reproductive choices and maternal care precludes informed autonomous decision-making	Risk that pregnant women do not receive enough counselling on the safety of a vaccine to make a proper risk assessment
Risks related to the effectiveness of sexually transmitted disease epidemic control strategies		Risk that a control strategy for an epidemic includes an unrealistic degree of abstaining
Risks related to the effectiveness of mother-to-child transmission programmes		Risk that prevention of mother-to-child transmission programmes are not accompanied by adequate counselling

**Table 3.** Identified proposed management strategies

Themes	Codes	Proposed management strategies (examples; full list as supplementary material)
Risks of misjudgments related to systematic uncertainty in policy decision-making and guideline development	Risks related to the appropriate design of individual hospital preparedness plans	Determining preemptive, transparent, and ethically sound preparedness, distribution and triage plans
	Risk related to lack of answers on normative questions	Loosening any necessary restrictions on individual rights as soon as the epidemic is over
Issues of harm affecting pregnant women	Issues of increased harm (mortality, morbidity) caused by insufficient access to health services in epidemics	Avoiding 'first come, first serve'procedures and distributing services randomly among equally prioritized groups
	Risks of increased harm (mortality, morbidity) caused by inadequate provision of health services in epidemics	Facilitating the use and distribution of unlicensed antivirals for patients affected by resistant strains
	Risk of harming infected mothers through stigmatization and criminalization	If mother-to-child transmission is identified: considering the possibility that some pregnancies are not intended
	General risks of harm caused by pregnancy in epidemics	Recognizing special risks for pregnant women in the epidemic and prioritizing resources accordingly
Risks of harming women of reproductive age	Increased risk of infection for women of reproductive age	Accepting donations to assisted reproductive technologies only from seronegative individuals
Issues of harming the child		Close collaboration between obstetrics and neonatology to optimize both maternal and neonatal outcomes
Issues of harming healthcare professionals		Providing vaccines to healthcare workers
Issues regarding pregnant women's autonomous decisions being compromised	Risks of medical factors compromising autonomy	Seeking and respecting advance directives that state preferences about end-of-life care and the fetal outcome
	Issues of direct interference by others	Liberalizing abortion laws and regarding some infections as sufficient grounds for termination of pregnancy
	Issues of indirect interference	Communicating the papal stance that the protective value of contraceptives can legitimize their use
	Risks that inadequate counselling about reproductive choices and maternal care precludes informed autonomous decision-making	Providing pregnant women with counseling and easy-to-understand information on vaccines and treatment options
Risks related to the effectiveness		Promoting faithfulness and partner reduction

## SUPPLEMENTARY MATERIAL

Supplementary Material 1: Reviewed papers are available at http://www.e-epih.org/.

Supplementary Material 2: Table S1 is available at http://www.e-epih.org/.

Supplementary Material 3: Table S2 is available at http://www.e-epih.org/.
