# Supplementary material for: Rapid qualitative review of ethical issues surrounding healthcare for pregnant women or women of reproductive age in epidemic outbreaks
Source: Epidemiol Health. 2018 Jan 23;40:e2018003. doi: 10.4178/epih.e2018003 (PMC5900442; doi:10.4178/epih.e2018003)
Supplement: Supplementary file 1 [file epih-40-e2018003-supplementary1.pdf]

**Supplementary Material 1**

**Reviewed papers**


1. Committee on Ethics. Committee opinion: no. 563: ethical issues in pandemic influenza planning concerning pregnant women. Obstet Gynecol 2013;121:1138-1143.
2. Bayer R. AIDS and the ethics of public health: challenges posed by a maturing epidemic. AIDS 1988;2 Suppl 1:S217-S221.
3. Bayer R. AIDS and the future of reproductive freedom. Milbank Q 1990;68:179-204.
4. Beigi R, Davis G, Hodges J, Akers A. Preparedness planning for pandemic influenza among large US maternity hospitals. Emerg Health Threats J 2009;2:e2.
5. Beigi RH, Hodges J, Baldisseri M, English D; Magee-Womens Hospital Ethics Committee. Clinical review: Considerations for the triage of maternity care during an influenza pandemic--one institution's approach. Crit Care 2010;14:225.
6. Benagiano G, Carrara S, Filippi V, Brosens I. Condoms, HIV and the Roman Catholic Church. Reprod Biomed Online 2011;22:701-709.
7. Berger BE, Omer SB. Could the United States experience rubella outbreaks as a result of vaccine refusal and disease importation? Hum Vaccin 2010;6:1016-1020.
8. Berger JT. Imagining the unthinkable, illuminating the present. J Clin Ethics 2011;22:17-19.
9. Black BO. Obstetrics in the time of Ebola: challenges and dilemmas in providing lifesaving care during a deadly epidemic. BJOG 2015;122:284-286.
10. Evans HE. Public policy and AIDS. Clin Perinatol 1994;21:29-38.

11. Farrell RM, Beigi RH. Pandemic influenza and pregnancy: an opportunity to reassess maternal bioethics. Am J Public Health 2009;99 Suppl 2:S231-S235.

12. van der Veen Y, Hahné S, Ruijs H, van Binnendijk R, Timen A, van Loon AM, et al. Rubella outbreak in an unvaccinated religious community in the Netherlands leads to cases of congenital rubella syndrome. Euro Surveill 2005;10:E051124.

13. Ibañez XA. Abuses of women's rights in sexual and reproductive health-care settings. HIV AIDS Policy Law Rev 2008;13:82-83.

14. Kisekka MN. AIDS in Uganda as a gender issue. Women Ther 1990;10:35-53.

15. Moukarram H, Nargund A, Photiou A, Kiran TS. Awareness and acceptance of the pandemic influenza (H1N1v 2009) vaccination among antenatal patients in a district general hospital. J Obstet Gynaecol 2012;32:537-539.

16. Nokleby H, Nicoll A. Risk groups and other target groups - preliminary ECDC guidance for developing influenza vaccination recommendations for the season 2010-11. Euro Surveill 2010;15:19525.

17. Pietrzak-Franger MM, Holmes MS. Disease, communication, and the ethics of (in) visibility. J Bioeth Inq 2014;11:441-444.

18. Requejo JH, Bhutta ZA. The post-2015 agenda: staying the course in maternal and child survival. Arch Dis Child 2015;100 Suppl 1:S76-S81.

19. Sacks V. Women and AIDS: an analysis of media misrepresentations. Soc Sci Med 1996;42:59-73.

20. Schenker JG. Report of the FIGO Committee for the Study of Ethical Aspects of Human Reproduction. Int J Gynaecol Obstet 1997;57:333-337.

21. Sher R. The role of women in the AIDS epidemic. Med Law 1993;12:467-469.

22. Wanamaker DA. From mother to child...a criminal pregnancy: should criminalization of the prenatal transfer of AIDS/HIV be the next step in the battle against this deadly epidemic? Dickinson Law Rev 1993;97:383-409.

23. Weiss K. Vaginal cancer: an iatrogenic disease? Int J Health Serv 1975;5:235-251.
