# Supplementary material for: Rapid qualitative review of ethical issues surrounding healthcare for pregnant women or women of reproductive age in epidemic outbreaks
Source: Epidemiol Health. 2018 Jan 23;40:e2018003. doi: 10.4178/epih.e2018003 (PMC5900442; doi:10.4178/epih.e2018003)
Supplement: Supplementary file 2 [file epih-40-e2018003-supplementary2.pdf]

Supplementary Material 2

Table S1. List of issues with quotations

**Themes**	**Codes**	**Subcodes**	**Quotation**
Risks of misguided judgement related to systematic **uncertainty** in **medical** decision-making	Risks related to **diagnosis** in pregnant women	Risk that an epidemic is asymptomatic and thus is hard to detect	“Rubella surveillance is also complicated by the fact that up to half of rubella cases may be asymptomatic. A recent article by Pandolfi, et al. cited difficulties with obtaining accurate data on CRS incidence, including weaknesses in surveillance systems, asymptomatic cases of rubella in pregnancy, variations in the clinical signs of CRS, and late presentation of CRS symptoms.” (Berger and Omer 2010)
		Risk that medical histories of pregnant women are not or inadequately reported to the treating physician	"Furthermore, the history is occasionally not reported correctly out of fear by patients or their relatives (for example, if they had been to a traditional birth attendant before coming, or if they have attempted to terminate the pregnancy)." (Black 2015)
		Risk that pregnancy complications give similar symptoms as the epidemic (e.g., EVD)	“In obstetrics there is a large crossover between the presentation of women with pregnancy complications and the alert symptoms for a suspected Ebola case. Spontaneous miscarriage, bleeding (including vaginal bleeding), abdominal pain, chest pain, joint pain, vomiting, stillbirth/intrauterine fetal death (IUFD), and fever are routinely encountered in isolation or combination as part of obstetrical referral criteria. The same symptoms can also form part of the ‘case definition’ for an Ebola alert, in particular when combined with a history of contact with Ebola (including suspicion) or attendance at funerals.” (Black 2015)

		Risk that tests deliver a high rate of false negatives, including cases where pregnant women are negative for the disease in her blood but not in amniotic fluid and placenta, hence continue to be contagious	"The pre-emptive testing of all patients has been suggested; however, this would be artificially reassuring because of high false-negative rates in the asymptomatic, incubating, or recovering patients, where viral loads may be low but not absent. Furthermore the polymerase chain reaction in a pregnant woman who has survived Ebola, and is negative for the virus in her blood, can remain positive with high viral loads in the amniotic fluid, placenta and fetus and hence continue to be contagious." (Black 2015)
	Risks related to **prevention and treatment** of infected pregnant women	Risk that transmission cannot effectively be prevented because the epidemic is most contagious before it becomes symptomatic	"The effectiveness of both Canadian and Dutch public health advice in preventing spread of rubella and in preventing pregnant women from becoming infected is probably limited. [...] Secondly, rubella is most infectious prior to the onset of rash (usual range one week prior to four days post rash onset). Finally, only a minority of cases are diagnosed since rubella virus infection can be asymptomatic in up to 50% of cases and, if symptomatic, usually has a mild course." (Hahné et al. 2015)
		Risk of lacking standards or guidance on when an investigational drug can and/or should be used in pregnant women	"Although exceptions do exist, pregnant women are excluded from initial and some postmarketing stages of drug trials principally out of concern for the effects of the drug on the fetus. Consequently, many new drugs come to the market without robust safety and efficacy information for pregnant women and fetuses. This knowledge gap has important implications, because the physiological and anatomical changes that take place during pregnancy can result in unexpected and potentially harmful side effects for pregnant women and their fetuses." (Farrell and Beigi 2009)

Risks of misjudgements related to systematic **uncertainty** in **policy decision-making and guideline development**	Risks related to the appropriate design or lack of individual **hospital preparedness plans**	Risk that there is no distribution principle for a scarce resource needed by pregnant women	“This approximate five- to ten-fold discrepancy could place much larger strains on hospitals that would have to provide for an increased number of critically ill pregnant women simultaneously converging on medical facilities and requiring critical care resources, such as ventilators. Determination of an ethically sound triage process to delineate use of ventilators for this population is an important component of pandemic influenza preparedness planning and management.“ (Beigi et al. 2010)
		Risk that a hospital cannot develop preparedness and prioritization plans regarding pregnant women and newborns because of a lack of guidance or literature	“Delineating an ethical framework for prioritizing and rationing medical services during a pandemic was a major challenge commonly articulated by the hospitals surveyed. There is some literature that addresses ethical issues in rationing for the general medical population, however, none addresses rationing and/or prioritization for pregnant women and newborns specifically” (Beigi et al. 2009)
		Risk that one cannot anticipate women's treatment choices in epidemics, which complicates the development of effective preparedness plans	Health care providers will be faced with operationalizing recommendations from public health officials for the use of pharmacologic agents. Pregnant patients will then be faced with choices about the use of these agents, specifically, while weighing the benefits against the harms to themselves and their fetus by using (1) a conventional antiviral with uncertain maternal and fetal side effects and an uncertain efficacy profile, (2) an experimental agent that may be incompletely tested or untested with the hope of some benefit, or (3) no pharmaceutical agent. Currently, no literature is available about how patients will make these decisions or about how to facilitate adequate informed consent for this level of decision-making." (Farrell and Beigi 2009)

	Risk related to lack of evidence on **technical** aspects	Risk that there is limited evidence on the effects of a given epidemic on pregnant women and their fetuses	"The evidence for risk of complications from the 'old' seasonal influenza in otherwise healthy pregnant women was contradictory. With the pandemic influenza, however, they were one of the risk groups, though European data are as yet scarce." (Nokleby and Nicoll 2010)
		Risk that there is limited evidence on how to design effective preparedness and prioritization for the protection of pregnant women and their fetuses	"The medical literature also includes logistical guidance as to how to approach this dilemma for the general medical population but no such guidance exists for the pregnant population that considers their unique characteristics and needs." (Beigi et al. 2010)
		Risk that there is limited evidence on the effects of a given drug on pregnant women and their fetuses	"This void in guidance is important given the important differences between general adult medical patients and obstetric or neonatal patient populations. Unlike most general medical patients, obstetric and neonatal patients are likely to have an increased susceptibility to the infectious agent. Despite this heightened susceptibility, less robust data exist on dosing for both pregnant women and neonates with antimicrobials to combat these infectious diseases." (Beigi et al. 2009)

		Risk that pregnant women are insufficiently included in the research agenda, such that knowledge about effects of treatment and prevention strategies, including use of investigational drugs, is lacking	"Currently, the two antivirals recommended for frontline use for H5N1 include oseltamivir (Tamiflu, F. Hoffman–La Roche Ltd, Basel, Switzerland) and zanamivir (Relenza, GlaxoSmithKline, London, England). Animal studies have suggested that both drugs will likely cross the human placenta, and, in larger doses, have been shown to cause minor skeletal abnormalities in laboratory animals. Much remains unknown about their use in pregnant humans. Label information for both agents discloses that well-designed clinical trials have not been performed among pregnant women." (Farrell and Beigi 2009)
		Risk that there is limited evidence about transmission risks for healthcare professionals, complicating, e.g., the balancing of risks for healthcare professionals with benefits of their services for patients	"Individuals without symptoms of Ebola are not thought to be contagious in normal circumstances; however, when considering obstetrics procedures alongside the high personal and public cost of occupational infection, there is insufficient evidence to fully balance the exposure risks (for example if a patient were to exhibit symptoms shortly after a caesarean section)." (Black 2015)
	Risk related to lack of guidance/answers on **normative** questions	Risks related to unclarity to what extent policies for the prevention of mother-to-child transmission may influence or interfere with women's reproductive choices	"Any program of prevention [of birth of infants with HIV] will require systematic attention to the following ethical and social questions: [...] For those women who are positive, what should be the nature of counselling? Should it be non-directive as in the genetic counselling model? Or ought the clinician/counsellor encourage the infected woman to refrain pregnancy? Will such directive counselling inevitably take on an element of coercion? For pregnant infected women, should counsellors encourage abortion? Is there a danger that such encouragement will take on elements of coercion?" (Bayer 1988).
		Risks related to unclarity on which rights the fetus has at which points in its development	"How should fetuses be considered among priorities for care? Does the degree to which they are viable matter, and, if so, how? Should limited resources be apportioned to save only fetuses who are expected to survive with minimal or no medical supports?" (Berger 2011)

		Risks related to relevance and ambiguities of the criterion of viability of the fetus	"Should resources be expended for all fetuses deemed viable under a hypothetical state of health system normalcy?" (Berger 2011)
		Risk related to uncertainty about the extent of obligations of public institutions towards healthcare professionals	"Another particular vulnerability is that of the clinicians, whose otherwise average personal medical risks for harm in catastrophe are multiplied by their professional commitments. These persons may find themselves exposed to contagious diseases and perilous work environments as they fulfill their role-related obligations. What degree of personal risk is acceptable? What is reckless? What do hospital administrations and local, state, and federal governments owe these persons in terms of mitigating risk of harm?" (Berger 2011)
Issues of harm affecting **pregnant women**	Issues of increased harms (mortality, morbidity) caused by insufficient **access** to health services in epidemics	Risk that because a woman is infected, she experiences difficulties accessing health services	"HIV-positive women are sometimes denied access to reproductive health services; or, if they do have access, they may be mistreated by the health care providers. Many providers reprimand them for bearing children, turn them away from public health care facilities, and refer them to private facilities specializing in HIV/AIDS (where costs are usually higher)." (Ibanez 2008)
		Risk that disparities in access to services are exacerbated during an outbreak	"Historical and recent experiences demonstrate that health disparities and barriers to access continue to effect disproportionate outcomes in pandemic influenza. These relate most directly to the effects of poverty and the maldistribution of resources." (Committee on Ethics of the American College of Obstetricians and Gynecologists 2013)

		Risk that during an epidemic, scarce resources are hoarded in order to avoid runouts, while these resources are urgently needed by pregnant women	"During a public health emergency, access to care and resources should be based on women's clinical needs rather than the type of insurance, if any, that they have or their prior relationship to a clinic or health care institution. Scarce resources should not be hoarded by health care institutions but should be distributed according to national or professional society guidelines." (Committee on Ethics of the American College of Obstetricians and Gynecologists 2013)
		Conflict of priority setting among pregnant women who consume a lot of resources in epidemic outbreaks and other, less vulnerable groups	"[Vulnerability] can result in a disproportionate level of injury across that population in addition to the consumption of a significant share of resources by a small segment of the population." (Farrell and Beigi 2009)
		Risk that prevention of mother-to-child transmission is focused only on the well-being of the infant, not on the mother	"The traditional focus of PMTCT programs as prevention programs for infants ignores the needs of women living with HIV/AIDS. The result is that often pregnant women cannot access antiretroviral drugs." (Ibanez 2008)
		Risk that triage plans unintentionally disadvantage pregnant women because they prioritize comorbidities which pregnant women normally do not have	"National literature regarding disaster planning suggests using a severity index to triage patients presenting to the hospital to determine who may be good candidates to receive the limited resources. These severity indices take the presence of significant comorbidities into consideration and/or seek to identify patients whose situations are potentially clinically futile. Although this approach will likely be very effective for older patients with significant comorbidities, it is doubtful that a large percentage of maternity patients will have significant comorbidities. Therefore, many will not 'screen out' of the first round of resource triage, resulting in a significant percentage of maternity patients still requiring decisions on how to prioritize the limited resources." (Beigi et al. 2009)

		Risk that triage plans disadvantage pregnant women	"Other data suggest that physicians’ ability to prognosticate vary widely by demographic characteristics and specialty, which introduces a potential source of bias in triage that may need to be managed." (Berger 2011)
		Risk that the needs of pregnant women cannot be addressed because personnel is itself adversely affected by the epidemic	"If healthcare workers were to become infected with Ebola the knock-on effect may be the closure of the unit itself." (Black 2015)
		Risk that healthcare professionals cease to provide key services which they perceive as putting them at risk of becoming infected	"The workers are, for example, gripped with mounting anxiety of contracting AIDS from patients through accidental pricking with needles or contact with contaminated blood through other means. For example, a nurse-midwife who runs a maternity home located ten kilometres from Masaka town and who had just returned from a visit in Britain, explained that she was no longer prescribing injections and that she was contemplating closing down the maternity home as it was located in an AIDS epidemic area." (Kisekka 1990)
		Risk that criminalizing mother-to-child transmission will discourage women from seeking early diagnosis and treatment	"In addition, the threat of criminal sanctions may act as a disincentive to seeking early diagnosis and treatment. If knowledge of HIV infection is necessary to criminally transfer the disease, it is logical that the attitude "what I don't know can't hurt me" may prevail." (Wanamaker 1993)

		Risk that pregnant women do not access vaccines as they perceive them as unsafe	"All 200 women (100%) returned the questionnaire. Among them, 190 women (95%) were aware of the vaccine, but only 85 women (42.5%) said they would have it. The main reasons for refusal were concerns about risk to the baby (50 women; 43.5%); risk to self (47 women; 40.9%) and risk to baby and self (18 women; 15.6%)" (Moukarram et al. 2009)
		Risk that because screening for a given epidemic is mandatory, women decide not to attend antenatal care	"Requiring pregnant women to be tested for HIV can discourage them from seeking prenatal care." (Ibanez 2008)
		Risk that rumours and fear cause general skepticism towards health services and thus lead pregnant women to seek medical help too late	"It is well documented that high maternal mortality rates are often caused by consecutive delays in recognition, access, and receipt of treatment. Certainly, this remains a significant issue in the affected region. The rumours and fear that accompany Ebola have magnified these delays. Stories of patients being isolated and then dying has fed into conspiracy theories that health centres are actively injecting patients with Ebola, or outright murdering them, scaring the population away." (Black 2015)
	Risks of increased harms (mortality, morbidity) caused by inadequate **provision of health services** in epidemics	Risk that no adequate compassionate and end-of-life care for pregnant women is provided	“Prospective planning and the allocation of sufficient resources for compassionate care for the terminally ill are of moral significance that equals the just allocation of health-preserving and life-saving resources.” (Committee on Ethics of the American College of Obstetricians and Gynecologists 2013)
		Risk that healthcare professionals transmit a disease to previously uninfected pregnant women	"Vaccination of health care professionals not only protects them from infection but also protects patients by decreasing the transmission of influenza in health care settings and by maintaining the health care workforce." (Committee on Ethics of the American College of Obstetricians and Gynecologists 2013)

		Risk that during an epidemic, protective equipment makes it more difficult to deliver obstetric services safely	"Personal protective equipment (PPE) is the standard precaution used for all procedures in an Ebola epidemic. The most restrictive clothing is reserved for suspected or confirmed cases. The practicality of using PPE during surgery remains controversial. The combination of a difficult operation, heat, reduced sensation, and reduced vision under layers of PPE can render it a hazard to safety as well as a protector. For example, if goggles repeatedly become misted over there is a higher risk of sharps injury." (Black 2015)
		Risk that pregnant women are negatively affected by drug resistance	"Preparation for potential drug resistance should be included in any pandemic plan. Concerns about drug exposure are relevant given the propensity of influenza viruses to develop resistance to the available medications. Resistance to amantadine (Symmetrel, Endo Pharmaceuticals, Chadds Ford, PA) and rimantadine (Flumadine, Forest Pharmaceuticals, St Louis, MO) have developed among seasonal influenza strains to such an extent that these drugs are no longer recommended for use." (Farrell and Beigi 2009)
		Risk that infected and non-infected patients will be treated in the same care site, thereby increasing the risk of transmission	"Access to these specialized medical centers [handling pregnancy complications] will mean that both infected and uninfected patients may be sent the same health care facility. Policymakers must work to preemptively devise methods to deliver ethical and scientifically sound medical care in a way that meets the needs of all pregnant patients presenting for care." (Farrell and Beigi 2009)
		Risk that the health system is ill-equipped for providing safe and effective interventions to women of reproductive age in an epidemic outbreak	"One of the most visible adverse effects of Uganda's last fifteen years of political and economic turmoil was the virtual collapse of the health delivery system. […] Given the past and current devastated state of the economy, it is doubtful that even with the best of credentials, a significant number of private facilities could conceivably afford sustained utilization of sterilized instruments." (Kisekka 1990)

		Risk that due to more frequent hospital visits (e.g., because of pregnancy), women are particularly likely to be infected during an outbreak	"As women are often caretakers of the sick, and during pregnancy are more likely to have recently attended a health clinic, they are a high-risk group for exposure to, and transmission of, Ebola." (Black 2015)
		Risk that administered medication is not safe for pregnant women (e.g., antiviral agents for influenza)	"Currently, the two antivirals recommended for frontline use for H5N1 include oseltamivir (Tamiflu, F. Hoffman–La Roche Ltd, Basel, Switzerland) and zanamivir (Relenza, GlaxoSmithKline, London, England). Animal studies have suggested that both drugs will likely cross the human placenta, and, in larger doses, have been shown to cause minor skeletal abnormalities in laboratory animals. Much remains unknown about their use in pregnant humans." (Farrell and Beigi 2009)
	Risk of harming infected mothers through **stigmatization and criminalization**	Risk that mandatory testing and programmes for the prevention of mother-to-child transmission do not ensure confidentiality, causing stigma and discrimination	"Mandatory testing is often accompanied by violations of the right to confidentiality and privacy." (Ibanez 2008)
		Risk that infected women who transmit the infection to their child are perceived as guilty for their infection and the transmission	"Pregnant, HIV-positive mothers, as 'unfit mothers,' are also seen as out of control--having failed to protect themselves from HIV, having failed to prevent themselves from getting pregnant, and having often engaged in activities deemed inappropriate for would-be mothers. Thus, these notions of individual responsibility for health and self-control are reflected in AIDS discourses through the stigmatization of those perceived as incapable of controlling what goes into and comes out of their bodies.<br>It is also perhaps this notion of individual responsibility for health that is behind the categorization of some people with AIDS as 'guilty' and others as 'innocent'." (Sacks 1996)

		Risk that pregnant women are legally prosecuted for mother-to-child transmission (e.g., because it is considered battery, negligence, or intended harm)	"A better remedy, therefore, may be found in an action for negligence. If a woman engages in conduct known to transmit AIDS/HIV, then she arguably should know that she is at risk -of being infected with the disease. Such a woman may arguably have a duty to ascertain whether she is infected before engaging in behaviour which could result in pregnancy". (Wanamaker 1996)
		Risk that criminalizing women for mother-to-child transmission will lead to unsafe abortions	"It is questionable, however, whether a woman "intentionally...transfers blood" to her child during pregnancy if she did not intend to become pregnant. Moreover, the threat of this type of prosecution could force the woman to terminate her pregnancy to avoid knowingly transferring contaminated blood or body fluid. If the woman does not discover that she is HIV positive until after her pregnancy has progressed beyond the time in which abortion is legal or safe, she may be left with no option but to violate the law or risk her own safety." (Wanamaker 1993)
	**General** risks of harm caused by pregnancy in epidemics	Risk that pregnancy can aggravate a pre-existing infection with an epidemic	"About one in four maternal deaths are caused by pre-existing medical conditions such as malaria, diabetes, obesity and HIV, the health impacts of which can be aggravated by pregnancy." (Requejo and Bhutta 2015)
Risks of harming **women of reproductive age**	Risks of increased harms caused by (organization of) **screening** in epidemics	Risk that due to privacy breaches after screening, a woman of reproductive age experiences domestic violence	"Mandatory testing requirements may also put women at risk of physical abuse, abandonment, neglect or even ostracism by their husbands, partners or community." (Ibanez 2008)
		Risk that screening of high-risk female populations in order to avoid mother-to-child transmission will result in stigmatization of this population	"If programs of screening are directed at high-risk women, will they result in the stigmatization of poor Black and Hispanic women?" (Bayer 1988)
	Risk of **stigmatization and persecution**	Risk that public discourse singles out women as sources of transmission, when in fact males are drivers in transmission, too	"Characteristically, adolescent and adult women of fertile age are singled out as bearers of morality. African society characteristically focuses on women for problems of sexuality and fertility. Thus, women are perceived as the sources of sexually transmitted diseases and therefore the custodians of morality." (Kisekka 1993)

		Risk that an anxious or irritated public is successful in demanding radical steps taken against infected women, e.g., sterilization, forced quarantine	"Perhaps the most drastic reaction to the AIDS situation was highlighted in the Taifa Empya newspaper (Aug 19, 1987) in which Masaka town residents were reported as suggesting that as an epidemiological measure, the government should quarantine women related to people with AIDS, while men should be castrated!" (Kisekka 1993)
		Risk that criminalizing mother-to-child transmission makes a woman liable regardless of whether the pregnancy was intended	"Because the AIDS/HIV epidemic presents such a serious public threat, a state may be able to impose a duty on a woman to ascertain whether she has or could have AIDS before engaging in an act that may result in pregnancy, and rightly subject her to strict liability for her failure to do so. It is therefore conceivable that a criminal law that restricts a woman's right to procreate, in order to safeguard the health and safety of the unborn child as well as the public, could pass the strict scrutiny test and withstand a constitutional challenge." (Wanamaker 1993)
	**Increased** risk of **infection** for women of reproductive age	Risk that because healthcare professionals are traditionally female, transmission risks for healthcare professionals affect women disproportionately	"Women, as we know, are the traditional health providers in the home and also feature prominently in the alternative health professions as birth attendants and spiritual mediums. Moreover, in the modern health-care delivery system, women are over-represented in the treatment and care of people with AIDS as well as in the professions of midwifery and nursing. The point therefore must be made that AIDS has not only increased women's burdens in the care of the terminally ill, but also risk of infection." (Kisekka 1993)
		Risk of increased susceptibility of women to an epidemic because of a lack of (access to) education, health services, social rights, voice in sexual matters.	"The generally low status of women within the family and society makes them particularly susceptible to HIV infection, a social vulnerability. Women's vulnerability stems from a lack of equal access to education, information and health service, inferior social rights, little or no voice in sexual matters, including adherence to safer sex practices and the use of condoms." (Sher 1993)

		Risk that oestrogen and progesterone increase a woman's susceptibility to a given epidemic, e.g., HIV	"Both oestrogen and progesterone affect the immune system and therefore may influence the natural history of HIV infection." (Sher 1993)
		Risk that through reproductive technologies, women receive donations from infected individuals	"Assisted reproductive technology requires the elective donation of gametes, embryos or surrogate carriage of pregnancy. Because of the elective nature of this technology confidential counselling and testing can be done and inclusion of only those with negative HIV status is possible. To protect the interests of those at risk of unwanted exposure to HIV including the potential child, only seronegative individuals should be allowed to participate." (Schenker 1997)
		Risk that wide acceptance of a religious norm that prohibits use of contraceptive individuals at an increased risk of contracting a sexually transmittable epidemic disease	"Bovens believes that an HIV-discordant couple does not bring about any bad outcome through condom use because there is no disrespect for the generative function of sex. He concluded that 'there are no in-principle objections against the use of condom by HIV-discordant couples and that policies denying them access to condoms are indefensible', because HIV-discordant couples have a right to continue consummating their marriage with minimal risks." (Benagiano et al. 2011)
	Risks of **male violence** as a reaction to preventive measures	Risk that intra-marital sexual violence increases because the woman is perceived to fear contracting an epidemic from her partner	"On the other hand, other men are accusing their wives of provoking sexual aggression by refusing their husband's sexual advances because of fear that the husbands might have AIDS due to extra-marital sexual relations or as mere reprisals for husbands' past extra-marital relations." (Kisekka 1993)
		Risk that because males are advised to change sexual behaviour as part of a disease control strategy, intra-marital sexual violence increases, e.g., out of frustration with monogamy	"The AIDS situation has triggered other unexpected acts of marital sexual violence. In some quarters, men who had been used to constant extra-marital sex and who are now monogamous with their wives were reported to be increasingly sexually violent with their wives as a result of their frustrations." (Kisekka 1993)

Issues of harming **the child**	Issues of **infecting** the child with the epidemic disease	Risk that an infected newborn's life will be shortened and largely be spent in a hospital	"Perhaps the most innocent and tragic of all of the victims of this disease are the children who are born HIV positive. These children are forced to endure extensive hospitalization and intermittent attention from medical professionals, rather than the love and stimulation of a constant caregiver, often knowing only a hospital setting as "home" during their brief lives." (Wanamaker 1993)
		Risk that the epidemic contributes to infections, congenital disorders, disabilities, miscarriages, etc.	"Rubella infection during pregnancy can lead to birth defects in the newborn, particularly if infection occurs early in gestation. Prenatal rubella infection is associated with several negative outcomes, including preterm delivery, stillbirth, congenital deafness, microcephaly, patent ductus arteriosus, mental retardation and other impairments. Collectively, this array of disorders is known as “congenital rubella syndrome” (CRS)." (Berger and Omer 2010)
		Conflict between parental autonomy regarding rejection of vaccines and the wellbeing of the fetus	"Vaccine refusal is a critical factor contributing to pockets of low coverage. Individuals can refuse vaccination (and parents can refuse vaccination of their children) on the basis of religious and/or personal beliefs, depending on state policies regarding nonmedical exemptions to immunization" (Berger and Omer 2010)
		Risk of mother-to-child transmission through breastfeeding because no safe and/or affordable alternatives are available	"Breastfeeding: In societies where safe, affordable alternative methods of infant feeding are available, it may be unethical for an HIV infected mother to breastfeed her child. Where the risks of alternative infant feeding are high, the balance of risk to the infant may favor making breastfeeding ethically justified." (Schenker 1997)

	**General issues** arising as a consequence of the epidemic	Conflict between wellbeing of the mother and the child	"The willingness of public health officials to urge infected women not to become pregnant must also be understood in the light of an emerging trend of<br>seeking to compel pregnant women to undergo medical treatments in order to protect the lives of their fetuses (Nelson and Milliken 1988). One study reported on 21 cases in which court orders were sought to override maternal refusals of such therapy. Permission was granted in all but three cases. When the directors of maternal/fetal medicine fellowship programs were questioned, just less than one-half believed that women who defied medical advice, thus endangering their fetuses, should be detained for medical supervision. [...] For George Annas (1987, 1213) the instances of coercive treatment suggested an ominous turn: "The beginning of an alliance between physicians and the state to force pregnant women to follow medical advice for the sake of their fetuses." (Bayer 1990)
		Risk that unfounded fear of mother-to-child transmission deters women from breastfeeding	"Of equal relevance to women's reproductive rights is the controversy regarding the relationship between breast-feeding and transmission of the AIDS virus to suckling babies. It must be realized that whether the evidence is conclusive or not, the mere publicity of the debate will probably deter some high-risk mothers from breastfeeding. If this happens, it is feared to culminate in more cases of infant mortality due to malnutrition and decreased immunology to infant diseases." (Kisekka 1990)
		Risk that in case of a severe congenital malformation, futile medical interventions prevent the fetus from dying with dignity	"The Committee recognised that newborn infants with severe malformations have the right to be allowed to die with dignity, without inappropriate or futile medical intervention when it is the considered view of both the parents and their doctors that this course is in the child's best interest." (Schenker 1997)
		Risk that a child will be born to infected parents who will pass away or be unable to raise the child	"Additionally, when a child is born to a mother and father who will not be alive to provide the necessary care and support, 60 the child will become the financial responsibility of the taxpaying public." (Wanamaker 1993)

		Risk of no treatment being available to infected child upon birth	"Gaps in coverage are evident for family planning, care around the time of birth and for case management of childhood diseases such as malaria, pneumonia and diarrhoea—these gaps are alarming given the indisputable evidence of the birth day as the riskiest day for both mother and newborn, that these three infectious diseases account for the bulk of postneonatal deaths, and the protective effect of family planning on maternal and child survival due to improved birth spacing and reduction in higher-order and other high-risk pregnancies." (Requejo and Bhutta 2015)
Issues of harming **healthcare professionals**		Risk that an epidemic is particularly dangerous for obstetricians because of frequent exposure to body fluids	"Obstetrics is considered to be one of the highest exposure-prone medical specialties in any context. Healthcare workers, equipment, and the general environment will frequently be exposed to large quantities of blood, amniotic fluid, urine, and faeces. Healthcare staff are a high-risk group for Ebola transmission and mortality in all healthcare facilities: in this sense, obstetrics during an Ebola epidemic can be considered an extremely high-risk specialty." (Black 2015)
		Conflict between health care workers' rights to protect themselves vs. need for health care workers' services	"Another particular vulnerability is that of the clinicians, whose otherwise average personal medical risks for harm in catastrophe are multiplied by their professional commitments. These persons may find themselves exposed to contagious diseases and perilous work environments as they fulfill their role-related obligations. What degree of personal risk is acceptable? What is reckless? What do hospital administrations and local, state, and federal governments owe these persons in terms of mitigating risk of harm? What do clinicians owe the populace by virtue of the specialized knowledge that they have acquired with public support? When should clinicians consider their own welfare, or their obligations to their families, above that of their patients?" (Berger 2011)

Issues of harming **the public/public health**		Risk that infected pregnant women act as a catalyst, transmitting the epidemic to other parts of the population	"[B]ecause of their health status, the medically vulnerable have a pivotal role in the morbidity and mortality of the entire population; they can serve as the catalyst for logarithmic spread of the infectious agent among the community." (Farrell and Beigi 2009)
		Risk that scarce resources are spent on pregnant women and subsequently lacking for the treatment of others	"[Vulnerability] can result in a disproportionate level of injury across that population in addition to the consumption of a significant share of resources by a small segment of the population." (Farrell and Beigi 2009)
		Risk that orphans who were born to infected parents cause costs for the taxpaying public	"Additionally, when a child is born to a mother and father who will not be alive to provide the necessary care and support, 60 the child will become the financial responsibility of the taxpaying public." (Wanamaker 1993)
		Conflict between reproductive and treatment choices of infected individuals vs. public interest in minimizing infection rates	"Taking cognizance of the human and particularly the African desire to immortalize oneself, it is probable that some infected individuals may strive to reproduce in order to leave a survivor after their own imminent deaths. Certainly, all these issues raise complex legal and ethical factors for Ugandan women which can only be solved or at least reduced by a concerted program of counselling and related policy measures." (Kisekka 1990)
Issues regarding pregnant women's **autonomous decisions** being compromised	Risks of **medical factors** compromising autonomy or autonomous decisions	Risk that infection renders the pregnant women unable (at least temporarily) to make decisions	"Given that pregnant women and newborns are at increased risk of both seasonal and pandemic influenza-related morbidity and mortality and that a disproportionate number of them may die during these periods, issues related to death and dying for pregnant women and neonates, including formulation of advance directives, should be incorporated into pandemic planning. Strategies related to death and dying should include the formulation of advance directives by pregnant women, the role of government or institutional stockpiling of supplies for palliative care, and education for alternative caregivers in the provision of palliative and hospice care services and grief counselling." (Committee on Ethics of the American College of Obstetricians and Gynecologists 2013)

		Risk that women hesitate to marry and/or become pregnant because of fear of becoming infected	"There is a pervasive morbid feeling of fatalism or pessimism expressed, especially among single people, to the effect, that every eligible person is a potential source of contracting AIDS. Young people hesitate to get married as they fear that eligible people might already have been infected with the AIDS virus." (Kisekka 1990)
	Issues of **direct interference** by others	Conflict between protecting patient privacy and confidentiality vs. the wellbeing of other parties (e.g., sexual partners, treating healthcare professionals, the general public)	"HIV infection is a transmissible disease with profound social and psychological implications for the woman, her partner and her family as well as for the health care team and society. Its characteristics include a prolonged latent period, a very high morbidity and mortality and social stigma. In addition, there is as yet no vaccine or curative treatment. Vertical transmission from mother to fetus, or to infant via breastmilk may occur. The incidence of this transmission may be reduced by drug therapy.<br>2. These facts bring sharply into focus the ethical conflict between patient privacy and confidentiality and the need to protect the sexual partners, the health care team and the public from a fatal communicable disease." (Schenker 1997)
		Risk that there is systematic and de facto mandatory testing for an epidemic without informed consent	"Providing adequate and accessible HIV testing and counselling is the first step in enabling HIV- positive women to fully exercise their reproductive rights. HIV testing must be grounded in an approach that protects human rights and respects ethical principles — meaning that it should be confidential, accompanied by counselling and only done with informed consent.<br>However, there is a growing trend to make testing mandatory. For instance, Kenya has specific regulations requiring informed consent, but only half of its public health facilities and 15 percent of its maternity facilities follow them." (Ibanez 2008)

		Risk that infected women's liberty is restricted in an epidemic (e.g., by quarantine)	"Perhaps the most drastic reaction to the AIDS situation was highlighted in the Taifa Empya newspaper (Aug 19, 1987) in which Masaka town residents were reported as suggesting that as an epidemiological measure, the government should quarantine women related to people with AIDS, while men should be castrated!" (Kisekka 1993)
		Risk that a pregnant woman's choices about diagnosis and/or treatment are not respected	"Historical precedent fosters real concern for the potential of a woman's interests and rights to become inappropriately diminished during pregnancy. Documented cases describe the ways in which competent pregnant patients' informed decisions to decline invasive interventions have been overridden by medical and legal establishments". (Farrell and Beigi 2009)
	Issues of **indirect interference**	Risk that social, cultural or religious norms pressure women with regards to family planning decisions in epidemics	"HIV-positive women are sometimes denied access to reproductive health services; or, if they do have access, they may be mistreated by the health care providers. Many providers reprimand them for bearing children, turn them away from public-health care facilities, and refer them to private facilities specializing in HIV/AIDS (where costs are usually higher)." (Ibanez 2008)
		Risk that because of a criminalization of mother-to-child transmission, an infected woman is effectively forced seek an abortion if pregnant	"If the act of transferring AIDS/HIV during pregnancy were criminalized, abortion would become the only alternative available to an HIV positive woman who becomes pregnant, despite the use of birth control, and does not want to risk criminal prosecution." (Wanamaker 1993)
		Risk that criminalizing mother-to-child transmission interferes with the right of the infected woman to freely practice religion (e.g., one that does not permit the use of contraceptives)	"A second constitutional argument against such legislation is that it burdens an individual's right to freely practice religion. A woman whose religious beliefs do not permit the use of contraceptive measures to prevent pregnancy or prohibit abortion of an unwanted or unplanned pregnancy would essentially be forced to choose between engaging in sexual relations at the risk of breaking the law, refraining from sexual activity, or abandoning her religious values." (Wanamaker 1993)

		Conflict between a woman's wish to use contraceptives vs. religious values which do not allow them	"As already mentioned, the position of the Roman Catholic Church on contraception has clearly influenced this prohibition, implying for instance that the use of condoms by HIV-discordant couples is illicit because of its contraceptive effect." (Benagiano et al. 2011)
		Risk that for an infected woman, access to medical services is conditional upon sterilization	"In many instances, consent to sterilization is not free and informed. Health care providers do not give women full information about the risk of mother-to-child transmission and available treatments; or they make the provision of other reproductive health services conditional on the woman accepting sterilization." (Ibanez 2008)
	Risks that inadequate **counselling** about reproductive choices and maternal care precludes informed autonomous decision-making	Risk that counselling ignores the cultural and social contexts of reproductive choice	"Among the most forceful critics of the public health posture on HIV infection and pregnancy was Janet Mitchell, a black obstetrician and perinatologist. Centering her challenge on the failure of those counselling deferral of pregnancy to appreciate the cultural and social contexts of reproductive choice and on their failure to understand the very different ways in which professional, often white, counselors and poor, often black and Hispanic, women understood the meaning of relative risk, she has underscored the importance of procreation to the women in whose defense she has written. For intravenous drug-using women the counsel of restraint was portrayed as especially devastating, threatening to deprive them of what hope they had for a better life." (Bayer 1990)
		Risk that counselling regarding reproductive and treatment decisions is directive (e.g., by tacit introduction of standards for "suitability" of procreation) or even coercive	"Alert to the empirical research that has demonstrated the subtly directive content of counselling that guides choices despite the claim to neutrality, feminists have been sharply critical of the "unbalanced" and distorting information which limits the options available to women. Thus, they have argued, for example, that the emphasis within counselling upon the burdens of bearing a child with some congenital defect denies prospective parents the opportunity to make reproductive choices in the light of the possibility that such a child could be a source of fulfillment." (Bayer

			1990)
		Risk that pregnant women do not receive enough counselling on the safety of a vaccine to make a proper risk assessment	"When the women were asked about their source of information about the vaccine, 128 women (64%) said they received information from the NHS; 59 (29.5%) from media and 13 (7.5%) from various other sources (Table II). They were also asked who counselled them about the vaccine. In 21 women (10.5%), counselling was provided by an obstetrician; in 43 women (21.5%) by the GP; in 66 women (33%) by a midwife or nurse and in 70 women (35%) counselling was not offered." (Moukarram et al. 2012)
Risks related to the effectiveness of STD-**epidemic control** strategies		Risk that a control strategy for an epidemic includes an unrealistic degree of abstaining	"At any rate, it is important that the Catholic Church acknowledges the African reality and in particular the fact that, whereas it is self-evident that never having sex will significantly reduce the risk of contracting an STI, including HIV, abstaining from sex is not a choice that many women living in Africa (or, to that matter, in the developing world) have." (Benagiano et al. 2011)

		Risk that the control strategy relies on contraceptives while no adequate support, counselling or guidance is provided	"'In my remarks I was not making a general statement about the condom issue, but merely said, and this is what caused such great offense, that we cannot solve the problem by distributing condoms. Much more needs to be done. We must stand close to the people, we must guide and help them; and we must do this both before and after they contract the disease.' […] [T]he Pope reaffirmed his belief that condoms per se cannot solve the problem of the spread of STI, including HIV/AIDS. The way he explained his 2009 remarks now clarifies that 'distributing condoms' cannot solve the problem and 'much more needs to be done'. It is difficult not to agree with this statement." (Benagiano et al. 2011)
		Risk of insufficient attention to behavioural or cultural changes necessary to control the epidemic	"As pointed out by Stoneburner and Low-Beer, success seems attributable in good part to the ability to communicate facts about AIDS through social networks since, despite substantial condom use and promotion of biomedical approaches, other African countries have shown neither similar behavioural responses, nor HIV prevalence declines of the same scale. Green et al. (2006) believe that in Uganda a decline in multi-partner sexual behaviour was the change most likely associated with HIV decline, particularly when extensive promotion of 'zero grazing' (faithfulness and partner reduction) was involved." (Benagiano et al. 2011)
		Risk that contraceptives make individuals less careful (e.g., engage in riskier sex)	"He gave several reasons for this conclusion: first, a phenomenon called 'risk compensation' (when people think they're made safe by using condoms at least some of the time, they actually engage in riskier sex); second, the fact that people seldom use condoms in steady relationships because doing so would imply a lack of trust; third, the specific African reality, namely that it is ongoing multiple relationships that drive Africa's worst epidemics. He concluded that in Africa successful strategies are those that 'break up these multiple and concurrent sexual networks' (Green, 2009)." (Benagiano et al. 2011)

		Risk that control strategies (e.g., ABC - Abstain, Be Faithful, and Consistent; UNAIDS) get instrumentalized by religious institutions or governments	"Five years ago Sinding (2005), while acknowledging that abstinence for younger adolescents, faithfulness in marriage and condom promotion have a place in international HIV/AIDS programmes, has complained that 'by twisting the ABC concept important international voices – the US government and the Vatican, in particular – have made ABC controversial. The actions of these major political actors are not only regrettable; given their influence over millions of people around the world, they represent a serious setback to efforts to bring HIV/AIDS under control'." (Benagiano et al. 2011)
		Risk that an epidemic control strategy based on behaviour changes of males fails to protect women	"Many people have come to regard AIDS as a panacea for the male double sex standard. Yet others, men in particular, have redefined "safe sex" to include extramarital affairs with a married person. Thus they are unwilling to cultivate monogamy." (Kisekka 1990)
		Risk that women struggle to avoid infection because control strategies rely on socially/culturally unacceptable methods (e.g., condom use)	"AIDS discourses that emphasize personal responsibility deflect attention away from the social and economic contexts which may make it more difficult for some to avoid infection--by telling women to 'just' use condoms when women are not the ones who wear condoms, and when even suggesting condom use may be considered socially unacceptable in a variety of ways." (Sacks 1996)
		Risk that an epidemic control communication strategy addressing males is ineffective	"Apart from abstinence from high-risk sexual behaviors, the proper use of condoms is considered to be the best protection against infection from AIDS. But perhaps because this is one method that shifts the burden of sexual and reproductive responsibility from the woman to the man, it has in the past never been aggressively promoted and marketed." (Kisekka 1990)

Risks related to the effectiveness of **mother-to-child transmission programmes**		Risk that prevention of mother-to-child transmission programmes are not accompanied by adequate counselling	"Adequate counselling must be a precondition to women's participation in PMTCT programs. To ensure an informed decision, women must be counselled on the risks and benefits of taking antiretroviral medication; the likelihood of transmission of HIV to their fetus; and the possible pain, impact and side effects of the treatment." (Ibanez 2008)
		Risk that prevention of mother-to-child transmission policies fail to have deterring effect on individuals who want to become pregnant	"Practicality of enforcement and the realistic probability of any deterrent effect must also be considered. Because "[e]very person convicted of a criminal offense has demonstrated a willingness to violate a legal proscription,"' it may not be effective to use the criminal law as a deterrent to transmission of AIDS/HIV." (Wanamaker 1993)
