# Supplementary material for: Rapid qualitative review of ethical issues surrounding healthcare for pregnant women or women of reproductive age in epidemic outbreaks
Source: Epidemiol Health. 2018 Jan 23;40:e2018003. doi: 10.4178/epih.e2018003 (PMC5900442; doi:10.4178/epih.e2018003)
Supplement: Supplementary file 3 [file epih-40-e2018003-supplementary3.pdf]

Supplementary Material 3

Table S2. List of proposed management strategies with quotations

Themes	Codes	Proposed Management Strategies	Quotation
Risks of misjudgements related to systematic uncertainty in **policy decision-making and guideline development**	Risks related to the appropriate design of individual **hospital preparedness plans**	Determining preemptive, transparent, and ethically sound preparedness, distribution and triage plans	"This approximate five- to ten-fold discrepancy could place much larger strains on hospitals that would have to provide for an increased number of critically ill pregnant women simultaneously converging on medical facilities and requiring critical care resources, such as ventilators. Determination of an ethically sound triage process to delineate use of ventilators for this population is an important component of pandemic influenza preparedness planning and management." (Beigi et al. 2010)
		Formulating clear inclusion and exclusion criteria	"In order to be eligible for receipt of a limited resource, each woman must meet the inclusion criteria by having the following clinical circumstances: clinically confirmed and viable pregnancy (defined in Table 1); and clear, documented need for the limited resource (for ventilation, refractory hypoxemia, respiratory acidosis (pH <7.25), impending respiratory failure and/or evidence of inability to protect the airway; for other critical care resources, clinically apparent hypotensive shock that is unresponsive to fluid resuscitation and requires the use of vasoactive medications that cannot be given on regular hospital units." (Beigi et al. 2010)

		Having triage assessments be made by a reasonable number of clinicians	"The group should consist of approximately three senior clinical individuals working together to make sound clinical assessments and allocation. Three clinicians allows for a full vetting of the relevant clinical issues, provides both obstetric and critical care input, and allows for majority decision making in rare cases of controversy that cannot be easily resolved by referring to the pre-defined criteria in the document. A suggested working group includes an experienced obstetric clinician, an experienced critical care clinician, and a senior obstetrical and/or critical care nursing representative." (Beigi et al. 2010)
	Risk related to lack of answers on **normative** questions	Loosening any necessary restrictions on individual rights as soon as the epidemic is over	"When individual liberties are conceded, the respect and human dignity granted to individuals must be preserved to the greatest extent possible. Individual rights and autonomy should be restored as soon as the emergency has passed." (Farrell and Beigi 2009)
		Consulting legal texts on reproductive rights of women	"An ethical analysis of these issues will require a review of the social history of efforts to affect the incidence of the perinatal transmission of venereal and other infectious diseases as well as of genetic disorders; a consideration of the legal literature on the reproductive rights of women, especially in the face of 'fetal-maternal' conflicts" (Bayer 1988)
Issues of harm affecting **pregnant women**	Issues of increased harms (mortality, morbidity) caused by insufficient **access** to health services in	Providing services to pregnant women in an epidemic purely on a needs-basis	"During a public health emergency, access to care and resources should be based on women's clinical needs rather than the type of insurance, if any, that they have or their prior relationship to a clinic or health care institution." (Committee on Ethics of the American College of Obstetricians and Gynecologists 2013)

	epidemics	Avoiding 'first come, first serve' procedures and distribute randomly among equally prioritized groups	"In a true pandemic, College Fellows should respect public health decisions regarding fair allocation of resources. ’First come, first served,’ is not a fair distribution strategy, for example, because it perpetuates existing social inequities. In addition, strategies such as prioritizing based on patient’s birth dates or social security numbers are not truly random and can introduce bias. Allocation strategies among equally prioritized individuals must be random to be fair." (Committee on Ethics of the American College of Obstetricians and Gynecologists 2013)
		Prioritizing pregnant women who are beyond a previously determined cut-off point in gestation	"When all other clinical parameters for prioritization are equal and two pregnant women are in need of the same resource, consideration should be given to the gestational age of the pregnancy as a potential prioritization cutoff. In this particular situation, facility-specific data may aid in determining a ‘cut-point’ at which time viability (the ability to be clinically managed and live after intensive interventions) of neonates may be expected. Pregnancies beyond the point of fetal viability may potentially receive higher priority given the fetus now has the ability to survive (with resource allocation) ex utero." (Beigi et al. 2010)
	Risks of increased harms (mortality, morbidity) caused by inadequate **provision of health services** in epidemics	Facilitating the use and distribution of unlicensed AV for patients affected by resistant strains	"It is not unrealistic that experimental or newly developed antivirals and vaccines may be the only agents that are effective against a rapidly mutating form of the virus. In anticipation of this potentiality, the US Department of Health and Human Services has concluded that ‘state and local health departments should be prepared to distribute unlicensed antiviral drugs (if needed) under FDA’s Investigational New Drug (IND) provisions.’" (Farrell and Beigi 2009)

		Designing careful diversion strategies that avoid patient-to-patient transmission	"Diversion strategies will be used to direct individuals to designated facilities for the management of existing medical problems or infection to prevent the exposure of one group to another and to limit viral transmission. This strategy will be problematic if used among the pregnant population without modification. […] Access to these specialized medical centers will mean that both infected and uninfected patients may be sent the same health care facility. Policymakers must work to preemptively devise methods to deliver ethical and scientifically sound medical care in a way that meets the needs of all pregnant patients presenting for care." (Farrell and Beigi 2009)
	Risk of harming infected mothers through **stigmatization and criminalization**	If mother-to-child transmission is prosecuted: considering the possibility that a mother might not have known her status at the time of conception	"Because of the long incubation period of the disease, it may be difficult to establish the requisite intent. The mother may not have knowledge of her HIV infection during her pregnancy or at the time of transmission. While a woman who knows she has AIDS/HIV may have a difficult time convincing a jury that she did not realize the contagious nature of the disease, a woman who did not even know she was infected with the virus would have no reason to know, with substantial certainty, that she was transferring the disease to her unborn child." (Wanamaker 1993)
		If mother-to-child transmission is prosecuted: considering the possibility that some pregnancies are not intended	"It is questionable, however, whether a woman 'intentionally ...transfers blood' to her child during pregnancy if she did not intend to become pregnant." (Wanamaker 1993)

		If mother-to-child transmission is prosecuted: considering the possibility that the child need not remain infected with certainty	"Proposing a cause of action for battery or infliction of emotional distress, one commentator reasoned that the doctrine of parent-child immunity could be defeated by establishing that the act was intentional because the mother knew with substantial certainty that her conduct would transfer the antibodies to her child. However, this theory fails to recognize that although the antibodies of the mother are always passively transferred to the child during pregnancy, not all children who are born to HIV-positive mothers will continue to carry the antibody eighteen months after birth. In fact, more than half of these children will not be HIV-positive within two years after birth. Consequently, while the mother may know with substantial certainty that she will pass the AIDS antibody to her unborn child during pregnancy, she does not necessarily know with substantial certainty that the child will remain independently HIV positive. Rather, the odds are in favor of the child growing up healthy." (Wanamaker 1993)
	**General** risks of harm caused by pregnancy in epidemics	Advising pregnant women to avoid contact with infected individuals	"In addition, the provincial Ministry of Health for Ontario (Ontario Ministry of Health and Long Term Care) has given advice on isolation of cases, quarantining of contacts and travel restrictions. This also differs from the Netherlands, where the emphasis of public health advice is on advising pregnant women to avoid contact with rubella patients." (van der Veen et al. 2005)
		Recognizing special risks for pregnant women in epidemic and prioritizing resources accordingly	"Because morbidity and mortality during an influenza pandemic are disproportionately high among pregnant women, the ethical considerations of protecting the public's health justifies the objective of assigning priority to pregnant women." (Committee on Ethics of the American College of Obstetricians and Gynecologists 2013)

Risks of harming **women of reproductive age**	Increased risk of **infection** for women of reproductive age	Accepting donations to assisted reproductive technologies only from seronegative individuals	"Assisted reproductive technology requires the elective donation of gametes, embryos or surrogate carriage of pregnancy. Because of the elective nature of this technology confidential counselling and testing can be done and inclusion of only those with negative HIV status is possible. To protect the interests of those at risk of unwanted exposure to HIV including the potential child, only seronegative individuals should be allowed to participate." (Schenker 1997)
Issues of harming **the child**		Collaborating closely in obstetrics and neonatology to optimize both maternal and neonatal outcomes	"“When a decision is made to perform a premature iatrogenic delivery for maternal benefit it is suggested that it be done after consultation with the relevant neonatal ICU personnel to assure resources are available to manage the preterm neonate. Given similar vulnerabilities, it is likely that neonatal ICUs will also be simultaneously faced with limited resource decisions during an influenza pandemic; thus, ongoing daily collaboration between obstetrics and neonatology services is required in order to optimize both maternal and neonatal outcomes.” (Beigi et al. 2010)
		Including into preparedness plans not only resource allocation strategies, but also preemptive plans on the medical management of mother and fetus	"Preparedness plans addressing the needs of pregnant women must also incorporate more than just the allocation strategies applicable for the general population. In addition to vulnerability resulting from physical changes of pregnancy, the gravid state introduces additional considerations about the medical management of the mother and fetus." (Farrell and Beigi 2010)

		Having parents and doctors decide together whether or not a given intervention is still within the newborn's best interest, or whether medical care should be withheld	"The Committee recognised that newborn infants with severe malformations have the right to be allowed to die with dignity, without inappropriate or futile medical intervention when it is the considered view of both the parents and their doctors that this course is in the child's best interest. [...] The Committee considered active euthanasia to be ethically unacceptable even when it appeared to be in the best interest of the child. However, the withholding or withdrawal of medical care (e.g. artificial ventilation, antibiotics, naso-gastric feeding, supplemental oxygen) was justified in such circumstances, provided that comfort care, including the offer of oral feeds, warmth, love and respect was maintained." (Schenker 1997)
		If necessary, making safe and affordable alternatives available	"Breastfeeding: In societies where safe, affordable alternative methods of infant feeding are available, it may be unethical for an HIV infected mother to breastfeed her child. Where the risks of alternative infant feeding are high, the balance of risk to the infant may favor making breastfeeding ethically justified." (Schenker 1997)
Issues of harming **healthcare professionals**		Providing vaccines to healthcare workers	"Vaccination of health care professionals not only protects them from infection but also protects patients by decreasing the transmission of influenza in health care settings and by maintaining the health care workforce." (Committee on Ethics of the American College of Obstetricians and Gynecologists 2013)
Issues regarding pregnant women's **autonomous decisions** being compromised	Risks of **medical factors** compromising autonomy	Seeking and respecting advanced directives which state preferences about end-of-life care and to the fetal outcome	"Given that newborns are at increased risk of both seasonal and pandemic influenza-related morbidity and mortality and that a disproportionate number of them may die during these periods, issues related to death and dying for pregnant women and neonates, including formulation of advance directives, should be incorporated into pandemic planning." (Committee on Ethics of the American College of Obstetricians and Gynecologists 2013)

	Issues of **direct interference** by others	Instructing healthcare professionals to respect autonomous patient decisions	"In situations that involve decision making concerning treatment of pregnant women, pregnant women's autonomous decisions should be respected." (Committee on Ethics of the American College of Obstetricians and Gynecologists 2013)
		Liberalizing abortion laws and regarding some infections as sufficient grounds for termination of pregnancy	"States must liberalize their abortion laws. States with restrictive abortion laws must, at a minimum, regard HIV-positive status as grounds for a legal abortion." (Ibanez 2008)
		Offering sterilization to Infected women only together with adequate counselling	"Coerced sterilization of women living with HIV/AIDS, which is becoming more frequent in some regions of the world, is a violation of women's reproductive rights. […] In many instances, consent to sterilization is not free and informed. Health care providers do not give women full information about the risk of mother-to-child transmission and available treatments; or they make the provision of other reproductive health services conditional on the woman accepting sterilization." (Ibanez 2008)
		With regards to testing: overriding requirements for informed consent, counselling, and communication of results only if transmission risk is high	"While appreciating the importance of confidentiality and patient privacy, the ethical responsibility of individual patients to prevent harm to others still exists. Informed consent must be obtained prior to testing for HIV infection and communication of the resultant information. Every effort should be made through counselling to convince individual patients of their responsibility to others including the importance of allowing such information to be used to protect sexual partners and health care workers. If in spite of every effort, consent is not obtained and the risk of transmission is high in certain circumstances, with consultation, it may be justified to override patient confidentiality" (Schenker 1997)
	Issues of **indirect interference**	Continuing to care for pregnant patients who decline certain interventions	"Pregnant patients who decline vaccination or antiviral medications should continue to be supported with appropriate care options that honor their autonomous choices." (Committee on Ethics of the American College of Obstetricians and Gynecologists 2013)

		Communicating the papal stance that the protective value of contraceptives can legitimate their use	"For Benedict, Father Lombardi added, the heart of the matter was ‘of taking responsibility, of taking into consideration the risk to the life of the person with whom you are having a relationship: this is if you’re a woman, a man or a transsexual’ (Donadio, 2010).<br>This additional explanation is of fundamental importance because it removes a critical obstacle to the use of condoms: that they cannot be utilized because they have a contraceptive effect. If female prostitutes can use it, then it doesn’t matter whether it prevents conception, as long as it protects against STI and HIV/AIDS and, therefore, saves lives." (Benagiano et al. 2011)
		Communicating transparently (and not overstating) the odds of mother-to-child transmission	"Moreover, we must not forget that when a child is born to an infected mother the odds are in favor of the child being healthy. Consequently, deterring women with AIDS/HIV from becoming pregnant or coercing them to seek abortions could be considered an extreme and unnecessary course of action." (Wanamaker 1993)
		Focusing on messages of compassion rather than judgement	"It is remarkable that there is a virtual absence of moral condemnation by all these sources including religious institutions. Thus, the country's Catholic Medical Bureau enjoins tobaliga ("love faithfully") and stresses the message of compassion rather than judgment with reference to Luke 6, 36-37. Similarly the Protestant Medical Bureau emphasizes that "AIDS is deadly" and preaches monogamy in marriage while calling on Christians to treat people with AIDS according to Matthew (25: 31-46) with food, drink, clothing, shelter, nursing care and comfort." (Kisekka 1993)

		Communicating the papal stance that HIV-discordant couples may use contraceptives	"Bovens believes that an HIV-discordant couple does not bring about any bad outcome through condom use because there is no disrespect for the generative function of sex. He concluded that 'there are no in-principle objections against the use of condom by HIV-discordant couples and that policies denying them access to condoms are indefensible', because HIV-discordant couples have a right to continue consummating their marriage with minimal risks." (Benagiano et al. 2011)
	Risks that inadequate **counselling** about reproductive choices and maternal care precludes informed autonomous decision-making	Providing pregnant women with counselling and easy to understand information on vaccines and treatment options	"Healthcare centres should provide pregnant women with easy to understand information such as patient's information leaflets explaining the benefits and safety of the vaccine." (Moukarram et al. 2012)
		Discussing concerns about treatment options in light of the woman's social and cultural context	"In situations that involve decision making concerning treatment of pregnant women, pregnant women's autonomous decisions should be respected. Concerns about the effect of maternal decisions on the fetus should be discussed in light of relevant medical evidence and understood within the context of each woman's broad social network, cultural beliefs, and values." (Committee on Ethics of the American College of Obstetricians and Gynecologists 2013)
		If legal: counselling women on pregnancy termination options	"The World Health Organization (WHO) has stated that a pregnant women [*sic*] who tests HIV-positive should be counselled on the options of continuing or terminating her pregnancy in countries where abortion is legal." (Ibanez 2008)
		Providing specific counselling for women who discover their HIV status during pregnancy	"Specific counselling should be devised for women who discover their HIV status once they are already pregnant and are seeking ante-natal care." (Ibanez 2008)

		Providing non-directive counselling on the withdrawal of medical care from a newborn with severe congenital malformations	"Prior to discussing the possibility of withholding or withdrawing medical care, the medical team has a responsibility to fully investigate and document the status of the malformed infant and to counsel the parents on their baby's condition, prognosis and on the management options. […] 8. In counselling parents the doctor should be careful not to impose his or her own cultural and religious prejudices on those whose beliefs and practices may be different, bearing in mind the legal requirements of the country. When a doctor's beliefs prevent the disclosing of all the possible options to the parents, the doctor has a duty to refer them to a colleague who is able to do so." (Schenker 1997)
Risks related to the effectiveness of STD-**epidemic control** strategies		Promoting faithfulness and partner reduction	"Green et al. (2006) believe that in Uganda a decline in multi-partner sexual behaviour was the change most likely associated with HIV decline, particularly when extensive promotion of 'zero grazing' (faithfulness and partner reduction) was involved. […] Confirmation of the reality of Ugandan changes, Kirby (2008) examined seven different types of evidence and concluded that all seven produced consistent evidence that people in Uganda first reduced their number of sexual partners prior to or outside of long-term marital or cohabiting relationships, and then increased their use of condoms with non-marital and non-cohabiting partners. Kirby believes that first reducing the number of sexual partners and breaking-up sexual networks and then reducing the chances of HIV transmission with remaining casual partners by using condoms can be achieved and can dramatically reduce the sexual transmission of HIV in generalized epidemics." (Benagiano et al. 2011)

		Educating women that insisting on condom use is essential to their protection	"Although it is a male contraceptive, women contacts of men who engage in high-risk sexual behaviors have a vested interest in the use of condoms and need therefore to be enlightened to insist on its utilization as a prophylactic against AIDS, if not as a contraceptive." (Kisekka 1993)
		Increasing and monitoring usage of condoms in the population	"Thus, a more effective strategy should include increased condom promotion for groups at high risk, more rigorous measurement of the impact of condom promotion and more research on how best to integrate condom promotion with other prevention strategies (Hearst and Chen, 2004)." (Benagiano et al. 2011)
		Presenting condom usage as an act of responsibility, care and love in social marketing campaigns	"At the same time, at the individual level, the idea of considering condom use as an act of responsibility, care and love merits further exploration from a social marketing perspective. Within this context, condom promotion should seek to create a culture of responsible sex in which condom use is seen as a mechanism to promote responsible behaviour (Benagiano et al., 2000)." (Benagiano et al. 2011)
